# Unmet Financial Needs of People with Psychotic Disorders—A Cross-Sectional Study in People with Psychotic Disorders, Parents, Siblings, and Controls

**DOI:** 10.3390/jcm13195945

**Published:** 2024-10-06

**Authors:** Josephien L. Jansen, Jiasi Hao, Richard Bruggeman, Claudia J. P. Simons, Marieke Van der Pluijm, Janneke Koerts, Lydia Krabbendam

**Affiliations:** 1 Department of Clinical and Developmental Neuropsychology, University of Groningen, Grote Kruisstraat 2/1, 9712 TS Groningen, The Netherlands; r.bruggeman@umcg.nl; 2 Department of Epidemiology, University Medical Center Groningen, Hanzeplein 1, 9713 GZ Groningen, The Netherlands; j.hao@umcg.nl; 3 Psychosis Department, University Center for Psychiatry, University Medical Center Groningen, Hanzeplein 1, 9713 GZ Groningen, The Netherlands; 4 Department of Psychiatry and Neuropsychology, Mental Health and Neuroscience Research Institute, Maastricht University Medical Center, Maastricht University, Vijverdalseweg 1, 6226 NB Maastricht, The Netherlands; c.simons@maastrichtuniversity.nl; 5 GGzE Institute for Mental Health Care, Dr. Poletlaan 40, 5626 ND Eindhoven, The Netherlands; 6 Department of Psychiatry, Amsterdam University Medical Center, University of Amsterdam, Meibergdreef 5, 1105 AZ Amsterdam, The Netherlands; m.vanderpluijm@amsterdamumc.nl; 7 Arkin, Institute for Mental Health, Klaprozenweg 111, 1033 NN Amsterdam, The Netherlands; 8 Department of Clinical, Neuro- and Developmental Psychology, Vrije Universiteit Amsterdam, Van der Boechorststraat 7, 1081 BT Amsterdam, The Netherlands; lydia.krabbendam@vu.nl

**Keywords:** psychotic disorders, unmet financial needs, financial satisfaction, predictors

## Abstract

**Background**. Psychotic disorders have a strong negative impact on people’s lives, including their financial situation. This study aimed to examine differences in unmet financial needs between people with psychotic disorders, parents, siblings, and controls. Secondly, we aimed to examine whether family clustering contributes to unmet financial needs. Lastly, we aimed to examine to what extent demographic, economic, psychiatric, functional, and cognitive characteristics and substance use predict unmet financial needs in people with psychosis. **Methods.** Data from the first assessment of people with psychosis (*n* = 956), siblings (*n* = 889), parents (*n* = 858), and controls (*n* = 496) included in the Genetic Risk and Outcome of Psychosis study were used. Group differences were assessed with Kruskal–Wallis tests (aim 1), while a mixed-effects logistic regression analysis and explorative and confirmative ordinal logistic regression analyses were conducted for aims 2 and 3, respectively. **Results.** Twenty-four percent of people with psychotic disorders reported unmet financial needs. These levels of unmet financial needs were significantly higher than levels for siblings, parents, and controls. We found a negligible influence of (direct) familial clustering on unmet financial needs. Lastly, cannabis and tobacco use significantly and consistently predicted higher levels of unmet financial needs of people with psychosis. **Conclusions.** Relatively high levels of unmet financial needs occurred in a heterogeneous group of people with psychosis, especially when people used cannabis or tobacco. Unmet financial needs can have detrimental consequences for mental health, stigmatization, leisure time activities, and social engagement. Thus, it is pivotal to recognize unmet financial needs, especially combined with substance use, as a crucial stressor for people with psychosis.

## 1. Introduction

Psychotic disorders strongly affect people’s daily functioning, including educational [1], vocational [2], and social functioning [3]. Psychotic disorders also negatively affect people’s financial situation. The vast majority of people with psychotic disorders are unemployed [4]. They often rely on government benefits as their main source of income, resulting in relatively low annual earnings [5]. Furthermore, studies indicate problems with financial performance, i.e., the ability to perform everyday financial tasks, such as counting change or writing checks, in people with psychosis [6,7,8,9,10,11,12]. Lastly, one study indicates lower financial competence in people with psychotic disorders compared to community-dwelling controls [13], where financial competence includes financial knowledge and financial judgement [14]. Concomitantly, one study reports that a relatively large proportion of people with psychosis spend a major part of their income on addictive substances (i.e., tobacco, alcohol, cannabis [15]).

In addition to these more objective measures of financial functioning, limited research indicates that people with psychotic disorders also show a lower subjective evaluation of their financial situation [16,17,18]. Some studies report on financial strain, i.e., people’s subjective ability to pay for bills [19]. These studies suggest that older people with schizophrenia experience more financial strain than randomly selected matched controls [16,17]. In our previous exploratory study, we reported that the prevalence of financial dissatisfaction in a large cohort of people with psychotic disorders was more than 25% [18]. This percentage is three to four times higher than in the general Dutch population [20].

People with psychosis might evaluate their financial situation as poor because of limited spending power, overspending, reduced financial performance or competence, but it might also be related to familial factors. For example, the chronic financial strain associated with low socioeconomic status of origin (SES-O) is associated with an increased risk of psychosis [21]. Given that low SES-O is shared by people with psychotic disorders and their family members, this could result in a lower evaluation of the familial financial situation, compared to the general population. However, the causality between SES-O and psychosis is complex and the evidence is conflicting [22,23,24,25]. Other indications for familial factors relating to a lower subjective evaluation of one’s financial situation comes from extensive research showing financial burden in parents and siblings caring for someone with psychosis [26,27,28,29,30,31,32,33,34,35,36]. These studies show that family members’ financial burden is due to indirect costs (e.g., reduced working hours to meet caregiver demands) and direct costs (i.e., providing financial support to their relative, due to, e.g., financial dependency).

Besides these familial factors, individual characteristics of people with psychotic disorders might be associated with a lower evaluation of one’s financial situation. Our previous study suggested that cannabis and other substance use, in particular, were associated with higher levels of financial dissatisfaction, while demographic and psychiatric characteristics, global, and social functioning appeared to play only a marginal role [18]. However, broader research on predictors of subjective measures of financial functioning is necessary. Firstly, we were unable to control for possibly overlapping factors due to the descriptive design of our previous study. Secondly, data on participant’s income were unavailable in our previous study. Thirdly, despite the large cohort, it consisted of participants with a generally long illness duration from the Northern Netherlands, a region with a lower average SES compared to other Dutch regions [37]. Finally, cognition is never explored as a predictor of people with psychotic disorders’ subjective financial functioning, even though extensive research suggests cognitive impairments in this group (e.g., [38,39,40,41]). Furthermore, cognition impacts overall subjective functioning in people with psychosis [42,43].

In the current study, participants’ subjective financial evaluation was defined as the extent to which people report to have enough money to meet financial needs [44]. The study aim was threefold. Firstly, to examine differences in unmet financial needs between people with psychotic disorders, parents, siblings, and controls. We hypothesized that people with psychotic disorders, parents, and siblings would show higher levels of unmet financial needs compared to controls, with people with psychosis showing the highest levels of unmet financial needs. Secondly, we aimed to examine whether family clustering (i.e., people with psychosis and parents and/or siblings within the same family) contributes to unmet financial needs. Due to the conflicting evidence, we tested this exploratorily. Lastly, we aimed to replicate and expand our previous findings [18] by examining to what extent demographic, economic, psychiatric, and functional characteristics, substance use, and cognitive functioning predict unmet financial needs in people with psychotic disorders with a relatively short illness duration from various areas in The Netherlands and Belgium. In line with our previous study, we hypothesized that particularly substance use would predict unmet financial needs in this group, while demographic, psychiatric, and functional characteristics would play a more marginal role. Since, to the best of our knowledge, economic characteristics and cognition were never examined as predictors of unmet financial needs in people with psychosis, these were examined in an exploratory way.

## 2. Materials and Methods

### 2.1. Database and Study Population

Participants in this study were participants of the Genetic Risk and Outcome of Psychosis (GROUP; [45]) study. GROUP is a naturalistic cohort study that started in 2004. The study ran in four university psychiatric centers (i.e., Amsterdam, Groningen, Maastricht, and Utrecht) and affiliated mental healthcare institutions in The Netherlands and Belgium. Participants were recruited through clinicians working in regional psychosis departments or academic centers. Their caseload was screened for the following inclusion criteria: (1) age between 16 and 50 years, (2) a diagnosis of a non-affective psychotic disorder according to the *Diagnostic and Statistical Manual of Mental Disorders Fourth edition* (DSM-IV [46]), and (3) a good command of the Dutch language. Participants were asked for informed consent for contacting their siblings and/or parents. Inclusion criteria for siblings were: (1) age between 16 and 50 years, and (2) a good command of the Dutch language. Parents were included when they had a good command of the Dutch language. When siblings or parents met the inclusion criteria but had a lifetime psychotic disorder, they were included in the patient group. Controls were selected through a system of random mailings to addresses in catchment area of the cases. Inclusion criteria for controls were: (1) age between 16 and 50 years, (2) no lifetime psychotic disorders, (3) no first-degree family member with a lifetime psychotic disorder, and (4) a good command of the Dutch language. All participants signed informed consent during their first assessment. The study was approved centrally by the Ethical Review Board of the University Medical Centre Utrecht (Research no. 04-003; 6 April 2004), and locally by review boards of participating institutes. Data of participants’ first assessment were used in the present study when they (1) were ≥18 years, (2) had disclosed their levels of (un)met financial needs, and (3) had available data on at least one of the other outcome measures. Of the 1119 participants with psychosis, 956 were included in the current study (Supplementary Figure S1). Family members and controls (≥18 years old) were included when they disclosed their level of (un)met financial needs. Included were 889 of 1059 siblings, 858 of 920 parents, and 496 of 586 controls.

### 2.2. Measures

#### 2.2.1. Measures Recorded for All Participants

(Un)met financial needs: (Un)met financial needs were assessed with an item from the shortened World Health Organization Quality of Life Questionnaire (WHOQOL-BREF, Dutch version [44]), an instrument showing good reliability and validity in the study population [47]. Participants were asked if they felt they had enough money to meet their needs in the last two weeks, on a 5-point Likert scale ranging from not at all (1) to *completely* (5). The scores were reverse-coded, so that higher scores represented higher levels of unmet financial needs.

Demographic characteristics: The region in which people were assessed (Amsterdam/Utrecht/Groningen/Maastricht) was recorded. Participants were asked to state their age (years), sex (male/female), ethnicity (Caucasian/non-Caucasian), and educational level by selecting one of the following: none, practice-oriented (primary school to pre-vocational secondary school), intermediate (general high school to intermediate vocational education), and theory-oriented (higher vocational education and university). They were also asked to state their marital status (married or living together/not married or living together), and living situation (alone/with parent(s)/with partner or family/sheltered living/other).

Economic characteristics: Participants were asked to state their source of income (wages/benefits—illness invalidity/benefits—unemployment/benefits—pension/study grant/parents/no income/other), and their gross monthly income (no own income/minimal or below/above minimal and below modal/above modal).

#### 2.2.2. Measures Recorded for People with Psychosis

All measures described below were validated to be used in the study population, see [48,49,50,51] for each measure, respectively. With regard to the cognitive test battery, these tests were selected for inclusion in the GROUP study on the basis of established reliability and validity [45].

Psychiatric characteristics: Diagnosis, illness duration (years), and number of lifetime psychotic episodes were recorded. Psychotic symptoms were assessed with the Positive and Negative Syndrome Scale (PANSS), a validated and widely used instrument for the assessment of the severity of psychotic symptoms [48]. We included positive and negative symptoms (both 7 items, scores ranging from 7 to 49). Higher scores indicated higher symptom severity. Being in remission (PANSS scores < 3; yes/no [52]) was assessed. Lastly, the proportion of unmet needs of patients as indicated by clinicians or researchers (ranging from 0.0 to 1.0) from the Camberwell Assessment of Need (CAN [49]) was assessed, including an item of unmet financial needs (0 = no need, 1 = met need, 2 = unmet need).

Functional characteristics: The Symptom and Disabilities subscales from the Global Assessment of Functioning scale (GAF; APA [53]) were used. On each subscale, scores ranged from 100 (extremely high functioning) to 1 (extremely impaired).

Substance use: Substance use was assessed with items from the short version of the Composite International Diagnostic Interview [54]. Tobacco use was assessed with the number of units per day. Alcohol use was assessed with number of units per week. Cannabis use was assessed as the most intense use in the last year (none/less than weekly/weekly/daily). Lastly, lifetime use of other drugs (i.e., the use of stimulants, sedatives, opiates, amphetamines, cocaine, PCP, or other drugs) was assessed (yes/no).

Cognitive functioning: Cognitive functioning was assessed with a comprehensive neuropsychological test battery. For this study, we included the Word Learning Task (i.e., immediate recall, delayed recall, retention rate [55]), the Continuous Performance Test-HQ (CPT-HQ; CPT sensitivity index and CPT variability [56]), the Wechsler Adult Intelligence Scale (WAIS-III; Digit Symbol Substitution Test, Information, Calculation, and Block Design test [57]), and the Response Set-Shifting Task (i.e., reaction time cost index scores and proportional cost index scores; adapted from [58]). With these test scores, we calculated a weighted standardized composite score of general cognition using principal component analysis (PCA). Details on the assessment and scoring of the tasks, and the calculation of the composite score were similar to procedures in previous GROUP-studies [45,59,60], except that oblique rotation (i.e., direct oblimin) was used in the PCA due to dependence between variables [61] (p. 644).

### 2.3. Statistical Analyses

Due to multiple testing and the large sample sizes, we used a conservative *p*-value of <0.01 to reduce type I errors for all the above-mentioned analyses. In the confirmative analyses, predictors were considered significant if both the *p*-value was <0.01 and the bootstrap BCa CI did not include zero. The n per analysis could vary due to missing data. Analyses were conducted with Statistical Package for the Social Sciences for IMG statistics version 28.0 and RStudio Version 2023.03.0+386 (including lme4 and mlmhelpr packages; [62,63]). GROUP data release 8.0 was used for the analyses.

#### 2.3.1. Demographic Characteristics

Differences in demographic characteristics between people with psychotic disorders, parents, siblings, and controls were tested by one-way analysis of variance (ANOVA) and χ^2^ tests. If a between-group difference was found on the ANOVA, post hoc pairwise comparisons (i.e., Tukey’s tests) were conducted to examine which groups differed.

#### 2.3.2. Group Differences in Unmet Financial Needs (Aim 1)

The differences in unmet financial needs on the WHOQOL-BREF between the four groups were examined by Kruskal–Wallis tests. The effect sizes for these differences were indicated by η^2^ and interpreted as small (<0.06), medium (0.06–0.14), or large (>0.14; [64]). If we found a between-group difference on the Kruskal–Wallis test, we conducted post hoc pairwise comparisons (i.e., Dunn tests) to examine which groups differed. Effect sizes were indicated by Cliff’s δ [65], and interpreted as small (<0.15), medium (0.15–0.33), or large (>0.33).

#### 2.3.3. Meeting Financial Needs and Family Clustering (Aim 2)

To examine the effect of family clustering, Fisher exact tests were run to investigate the associations between unmet financial needs of (1) people with psychosis and siblings, and (2) people with psychosis and parents within the same family [66]. Effect sizes were indicated by Cramer’s V, and interpreted as small (≤0.10), medium (0.11–0.30), or large (>0.30; [67]). To further assess whether participants within the same family showed similar levels of unmet financial needs, we conducted a mixed-effects logistic regression with families as a random effect. Given the limitations in statistical power when conducting a multinomial mixed-effects logistic analysis, we dichotomized the outcome (i.e., unmet financial needs = not at all/almost not able to meet financial needs, met financial needs = average to completely able to meet financial needs) for this specific analysis. First, we ran an empty model. Then, multivariable models were run by including covariates that exhibited significant group differences in previous analyses. The final multivariable model was selected jointly by Akaike Information Criterion (AIC), Bayesian Information Criterion (BIC), and log-likelihood. The intraclass correlation coefficient (ICC) was calculated [62]. A log-likelihood ratio test was performed to compare models with and without the family random effect. The assumptions associated with the (mixed-effects) logistic regression analysis were tested.

#### 2.3.4. Predictors of Unmet Financial Needs in People with Psychosis (Aim 3)

For people with psychotic disorders, Spearman rho correlations and their 99% confidence intervals (CIs) were reported between unmet financial needs and the continuous demographic, psychiatric, functional, and cognitive characteristics and substance use (Supplementary Table S1). To examine to what extent demographic, psychiatric, functional, and cognitive characteristics and substance use predicted unmet financial needs in people with psychotic disorders, we performed exploratory and confirmatory analyses. Explorative analyses were performed to determine which of the above-mentioned independent variables could predict levels of unmet financial needs. The data were randomly divided into two equally large subsamples (i.e., samples A and B). Univariate ordinal logistic regression analyses (with listwise deletion) were performed in order to assess the predictive value of each independent variable on levels of unmet financial needs separately. In sample B, only significant predictors from sample A were included.

Next, a confirmative analysis was performed to determine the sustainability of the results from the explorative analyses. A multivariable ordinal logistic regression analysis (Enter method, listwise deletion) was performed on the total sample (sample A + B) using the significant predictors from the exploratory analyses. Bootstrapping with 1000 samples was used to derive bias-corrected accelerated (BCa [68]) 99% CIs for the regression coefficients. Effect sizes were indicated by Nagelkerke’s explained variance (*R*^2^) and interpreted as small (≤0.12), medium (0.13–0.25), or large (≥0.26 [69]). The assumptions associated with logistic regression analysis were tested. The variance inflation factor (VIF) was inspected for multicollinearity.

## 3. Results

### 3.1. Demographic Characteristics

Table 1 shows the demographic characteristics of the four groups. The groups differed significantly regarding region of assessment (χ^2 ^(9) = 79.56, *p* < 0.001). People with psychosis, siblings, and parents were more often assessed in Amsterdam and Groningen, whereas controls were more often assessed in Maastricht and Utrecht. There was an effect of age (F (3, 3195) = 2180.40, *p* < 0.001). Besides the expected age differences between parents and the other groups (all *p* < 0.001), controls were older than people with psychosis and siblings (both *p* < 0.001). There was an effect of estimated IQ (F (2, 3112) = 95.10, *p* < 0.001). Controls had significantly higher estimated IQs than parents, siblings, and people with psychotic disorders (all *p* < 0.001), and parents and siblings had significantly higher estimated IQs than people with psychotic disorders (*p* < 0.001). Furthermore, the four groups differed significantly regarding sex (χ^2 ^(3) = 273.81, *p* < 0.001) as 75.9% of people with psychotic disorders were male, whereas these proportions were smaller in all other groups. The groups also differed significantly regarding educational level (χ^2 ^(9) = 254.31, *p* < 0.001); people with psychosis more often had a lower educational level compared to the other groups. The groups also differed significantly in ethnicity (χ^2 ^(3) = 64.25, *p* < 0.001); the majority of people with psychotic disorders were Caucasian (79.1%), even more so for parents, siblings, and controls (>82%). There were significant differences regarding marital status (χ^2 ^(3) = 680.44, *p* < 0.001) and living situation (χ^2 ^(12) = 593.52, *p* < 0.001). People with psychosis were more often unmarried, living alone, with parent(s), or in sheltered living than the other groups. Lastly, the groups differed significantly regarding source of income (χ^2 ^(21) = 995.98, *p* < 0.001) and gross monthly income (χ^2 ^(12) = 556.11, *p* < 0.001). The main source of income of people with psychosis was more often illness invalidity benefits, compared to wages in the other three groups. In addition, the majority of people with psychosis had a (below) minimal gross monthly income, with the other groups having higher levels of gross monthly income.

### 3.2. Group Differences in Unmet Financial Needs (Aim 1)

**Figure 1:**
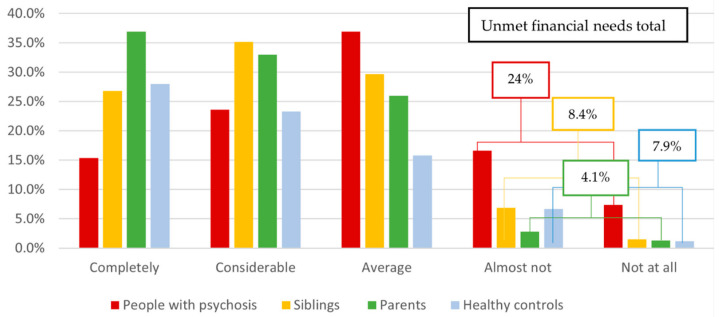
Group differences in unmet financial needs.

Figure 1 shows the levels of meeting financial needs of the four groups. On average, people with psychotic disorders felt *averagely able* to meet financial needs. However, 24% of people with psychosis reported (almost) not having enough money to meet their financial needs. This percentage was considerably higher than percentages of unmet financial needs as indicated by clinicians/researchers on the CAN (9.1%, n = 92). On average, siblings, parents, and controls felt *averagely* to *considerably* able to meet their financial needs, whereas only 4.1% of parents, 8.4% of siblings, and 7.9% of controls reported (almost) not having enough money to meet their financial needs. Indeed, a significant difference (medium effect size) was found between the four groups regarding meeting financial needs (H (3) = 263.63, *p* < 0.001; η^2^ = 0.08). People with psychotic disorders had significantly higher levels of unmet financial needs (medium–large effect sizes) compared to controls (δ = 0.32; *p* < 0.001), siblings (δ = 0.28, *p* < 0.001), and parents (δ = 0.39, *p* < 0.001). Siblings also had significantly higher levels of unmet financial needs (small effect size) compared to parents (δ = 0.13, *p* < 0.001) but not controls (*p* = 0.24). Lastly, parents did not differ from controls regarding levels of meeting financial needs (*p* = 0.01).

### 3.3. Meeting Financial Needs and Family Clustering (Aim 2)

A pairwise comparison of meeting financial needs of people with psychosis and siblings within the same family was not significant (Cramer’s V = 0.09, *p* = 0.04). A pairwise comparison of people with psychosis and parents within the same family was significant, with a medium effect size (Cramer’s V = 0.14, *p* < 0.001).

The assumptions associated with the (mixed-effects) logistic regression analysis were all met. The ICC estimated from the empty model suggested the existence of a familial effect, with 23% of the total variance in a person’s level of unmet financial needs attributed to variations between the families (Table 2; *p* < 0.001). However, in the multivariable model, controlling for covariates, family only contributed to 2.0% of the total variance in the outcome, and the significance disappeared (*p* = 0.82).

### 3.4. Predictors of Unmet Financial Needs in People with Psychosis (Aim 3)

The psychiatric, functional, and cognitive characteristics and substance use of people with psychotic disorders in the total sample (sample A + B) and the correlations with unmet financial needs are shown in Supplementary Table S1. Table 3 shows the results of the univariate ordinal regression analyses (i.e., exploratory analyses). The estimated IQ exhibited multicollinearity with the other dependent variables in the model (VIF > 10 [70]) and was excluded from the analyses. All other assumptions associated with the logistic regression analysis were met.

In sample A, 11 variables significantly predicted unmet financial needs (all small effect sizes), including region of assessment (Amsterdam, Groningen, and Maastricht vs. Utrecht), not being married/living together, source of income being “other” (vs. earning wages), higher positive symptoms, lower scores on indicators of global functioning (i.e., more symptoms and disabilities), more tobacco use per day, and less than weekly or daily cannabis use in the last year (vs. no cannabis use).

In sample B, 6 out of 11 predictors were significant (all small effect sizes; Table 3). Unmet financial needs were predicted by higher positive symptoms, lower scores on indicators of global functioning (i.e., more symptoms and disabilities), more tobacco use per day, and less than weekly or daily cannabis use in the last year (vs. no cannabis use). Region of assessment, marital status, and source of income were no significant predictors in sample B.

In the confirmatory analyses, the 6 significant predictors from the exploratory analyses were included in a multiple ordinal regression model (Table 3). The total model for unmet financial needs was significant and had a medium effect size, explaining 13% of the variance (*χ^2^* = 102.30, df = 7, *p* < 0.001). Individual predictors significantly predicting unmet financial needs included more tobacco use per day, and daily cannabis use in the last year (vs. no cannabis use).

## 4. Discussion

This study aimed to examine differences in unmet financial needs between people with psychotic disorders, parents, siblings, and controls. Additionally, we aimed to examine whether family clustering contributes to unmet financial needs, and to what extent demographic, economic, psychiatric, functional, and cognitive characteristics and substance use predict unmet financial needs in people with psychosis.

In line with our hypothesis, people with psychotic disorders showed significantly higher levels of unmet financial needs compared to parents, siblings, and controls. Almost one-quarter of people with psychosis reported unmet financial needs, compared to only 7.9% of controls, 4.1% of parents, and 8.4% of siblings. These findings are consistent with previous studies showing that people with psychosis have problems in objective financial functioning [4,5,6,7,8,9,10,11,12,13] and subjective financial functioning [16,17]. Particularly, the current findings align with our previous study, in which one-quarter of people with psychotic disorders reported financial dissatisfaction [18]. This percentage was three to four times higher than proportions of financial dissatisfaction in the general population [20]. In our previous study, people with psychosis had relatively long illness durations (mean > 13 years [18]) whereas in the current study, people’s illness duration was generally short (mean < 5 years), indicating that relatively high levels of financial dissatisfaction/unmet financial needs occur in people with psychosis in all phases of their illness. Knowingly, based on these findings, the causal direction could not be determined. On the one hand, research indicates that psychosis is a risk factor for financial problems [4,5]. On the other hand, negative financial conditions (e.g., low income or poverty) are recognized as risk factors for a worsening of mental health generally [71], and the development and maintenance of psychosis specifically [72]. Simultaneously, negative financial conditions are linked to higher stigma [73] and less social engagement [74] in people with severe mental illness. Irrespective of the direction of the relationship, these findings highlight that not having enough money to meet one’s financial needs is an important stressor for healthcare professionals working with people with psychosis to be aware of. Unfortunately, the discrepancy between unmet financial needs as indicated by people with psychosis and as perceived by clinicians or researchers indicates that this stressor is often overlooked.

Contrary to our expectations, parents and siblings did not significantly differ from controls regarding unmet financial needs. Furthermore, our results suggest a negligible effect of family clustering on meeting financial needs. This outcome is noteworthy, considering that previous studies consistently demonstrated a high financial burden on caregivers of people with psychosis [26,27,28,29,30,31,32,33,34,35,36]. In our study, family members were not necessarily caregivers. Yet, their involvement in their relative’s lives was implicit. It is important to note that an indirect familial effect could not be ruled out and warrants further exploration in future studies. The covariates contributing to the diminished familial clustering (i.e., ethnicity, educational level, and living situation) are often shared among family members, suggesting that some shared family-level factors can still play a role. However, these variables can also be influenced by other—individual—factors unrelated to family dynamics. For instance, a person might live with an intimate partner, or educational level can differ from that of family members due to the impact of psychotic symptoms. Thus, our results suggest that the individual characteristics or the condition itself, rather than SES-O or other shared familial factors, are key for unmet financial needs in people with psychotic disorders [73,75].

Indeed, individual characteristics that predicted higher levels of unmet financial needs in people with psychotic disorders in the exploratory analysis included demographic (i.e., region of assessment, not being married/not living together), economic (i.e., having another source of income as compared to earning wages), psychiatric (i.e., more positive symptoms), and functional characteristics (i.e., lower global functioning) and substance use (i.e., more tobacco and cannabis use). However, only tobacco and cannabis use remained significant predictors of unmet financial needs in the confirmatory analyses. Again, these findings are consistent with our previous study, in which cannabis and other substance users were significantly more often financially dissatisfied than nonusers, whereas other factors played a more marginal role. One possible explanation is the high expenditure associated with substance use in people with psychosis [15]. Additionally, substance use can complicate people’s financial management [76]. Together, our findings suggest that substance use, most consistently cannabis use, is an important factor to consider when people report low subjective evaluations of their financial situation. Furthermore, relatively high levels of unmet financial needs seem to occur in a heterogeneous group of people with psychosis, independent of demographic, economic, psychiatric, and functional characteristics. Unmet financial needs also appear to be independent of cognition, despite/contrasting with other studies reporting cognition affects other domains of subjective functioning in this group [42,43].

One possible mechanism behind relatively high levels of unmet financial needs in this group is that having a psychotic disorder leads to reduced educational and vocational opportunities, resulting in lower current SES (e.g., [22]). However, surprisingly, while in the current study, people with psychotic disorders indeed had lower educational levels, lower income levels, and higher proportions of illness invalidity benefits than the other groups, these factors did not consistently predict unmet financial needs. This finding suggests that the subjective evaluation of having enough money to meet one’s needs appears distinct from someone’s objective current SES. While this is counterintuitive, it is consistent with previous findings in the general population [77,78]. One possible explanation is that people with psychotic disorders can have substantial healthcare costs [36,79,80], which influences their disposable but not their gross income, which was measured in the present study. Additionally, other allocations of income, such as spending money on substance use [15], could create financial strain, without influencing gross income levels. Lastly, limited research suggests diminished financial competence in people with psychosis [13]. Financial competence (encompassing financial knowledge and financial judgment) is positively associated with subjective financial evaluations in the general population (e.g., [81]). Lower financial competence might lead to higher levels of unmet financial needs, irrespective of actual income levels. Thus, future studies are encouraged to include more detailed objective financial indicators (i.e., net income, disposable income, allocation of income, financial competence) as potential predictors of meeting financial needs in people with psychotic disorders.

A strength of the naturalistic nature of the current cohort study is that results can be generalized to people with psychosis in clinical practice. Furthermore, the study uniquely includes the subjective evaluations of family members, thereby expanding the limited scientific literature on the topic with evaluations of other important stakeholders. Some limitations need to be addressed when interpreting the results. Most importantly, unmet financial needs were assessed with one item from the WHOQOL-BREF [44]. To provide further insight in the association between psychosis and financial needs, future studies are recommended to use more detailed questions regarding having enough money to meet specific needs (e.g., nutrition, housing, leisure activities). In addition, we encourage including more detailed questionnaires related to this construct (e.g., assessing satisfaction with income and savings, financial stress). Secondly, we were unable to account for some potential confounders, which might affect financial outcomes. For example, research suggests that comorbid psychiatric conditions, such as anxiety or depression, are common among people with psychosis [82], while these conditions can also influence financial outcomes [83]. Thus, it is important to include information on comorbidities in future studies. Thirdly, it is essential to evaluate the results of this study in its social and economic context, as policies concerning social determinants of health, such as finances, and (the legality of) substance use vary across countries. Fourthly, we included data from participant’s first assessments, due to the large quantities of missing data in the subsequent waves (e.g., parents were not included in the follow-up assessments). This may limit generalizability. However, regarding group comparisons on unmet financial needs, data from the second and third assessments seem to correspond with our results. In these, the proportions of people with psychosis who cannot meet their financial needs are three to seven times higher than those of their siblings and controls (Supplementary Table S2). Lastly, a selection bias might be present. Participants who are willing and able to participate in lengthy studies may differ from participants in smaller studies or those who refuse or are ineligible to participate. Also, differences between family members and people with psychosis might be overestimated, if family members who are unable or unwilling to participate have some shared characteristics with their affected relative. Unfortunately, information on response rates was lacking.

These limitations notwithstanding, we conclude that one-fourth of people with psychotic disorders report unmet financial needs. This percentage is considerably higher than in siblings, parents, and controls. The negligible family effect on unmet financial needs highlights the distinctive challenge for people with psychosis. Lastly, cannabis and tobacco use consistently predict higher levels of unmet financial needs. Unmet financial needs appear to be relatively independent of demographic, economic, psychiatric, functional, and cognitive characteristics. Overall, our results suggest that relatively high levels of unmet financial needs occur in a heterogeneous group of people with psychosis. 

Unmet financial needs can have detrimental consequences on mental health, stigmatization, leisure time activities and social engagement. It is recognized that professionals should routinely consider financial issues in their contact with patients (for a review see [84]) including people with psychosis [15,85]. Despite the growing availability of interventions such as financial therapy, which integrates mental health and financial health [86,87], mental healthcare professionals often do not address these issues [88]. This is perhaps due to insufficient knowledge about social determinants of health, such as finances, and existing interventions. Other likely barriers include short consultation times or a focus on symptomatic recovery (e.g., symptom severity, side effects [85,89]). However, recognizing unmet financial needs as a crucial stressor, especially among people using substances, is a pivotal first step toward timely addressing their negative consequences for people with psychosis.

## Figures and Tables

**Table 1:** Demographic characteristics of people with psychotic disorders, siblings, parents, and controls.

	People with Psychosis	Siblings	Parents	Controls
N	956	889	858	496
Age M (SD) ^1^	28.2 (7.9)	28.7 (7.9)	54.8 (6.7)	31.5 (10.2)
Gender, % male (*n*)	75.9 (726)	45.4 (404)	42.9 (368)	44.0 (218)
Region of assessment, % (*n*)				
Utrecht	22.8 (218)	24.0 (213)	24.4 (209)	28.6 (142)
Amsterdam	27.3 (261)	25.9 (230)	28.0 (240)	18.1 (90)
Groningen	26.6 (254)	28.5 (253)	27.6 (237)	16.5 (82)
Maastricht	23.3 (223)	21.7 (193)	20.0 (172)	36.7 (182)
Estimated IQ, M (SD) ^2^	95.2 (16.5)	103.1 (15.6)	103.1 (17.1)	109.9 (15.1)
Ethnicity, Caucasian, % (*n*)	79.1 (739)	82.9 (735)	88.8 (756)	93.0 (452)
Educational level, % (*n*)				
None	0.9 (8)	0.1 (1)	0.5 (3)	-
Practice-oriented	42.7 (401)	22.7 (198)	30.4 (183)	13.1 (65)
Intermediate	42.7 (401)	44.2 (386)	32.2 (194)	48.1 (238)
Theory-oriented	13.7 (129)	33.0 (288)	36.9 (222)	38.8 (192)
Marital status, not married/living together, % (*n*)	90.7 (867)	57.3 (509)	-	58.9 (292)
Living situation, % (*n*)				
Single	35.1 (307)	21.8 (183)	8.3 (15) ^a^	23.9 (113)
With parent(s)	37.4 (327)	22.4 (188)	6.6 (12)	21.6 (102)
With partner/family	11.1 (97)	49.6 (416)	83.4 (151)	49.8 (235)
Sheltered living	9.8 (86)	0.01 (1)	-	-
Other	6.5 (57)	6.0 (50)	1.7 (3)	4.7 (22)
Source of income, *n*	733	610	601	404
Wages, % (*n*)	23.5 (175)	71.8 (437)	67.6 (406)	68.6 (277)
Benefits—illness invalidity, % (*n*)	34.1 (254)	3.0 (18)	5.7 (34)	1.0 (4)
Benefits—unemployment, % (*n*)	9.5 (71)	2.3 (14)	2.2 (13)	0.7 (3)
Benefits—pension, % (*n*)	2.3 (17)	1.0 (6)	14.8 (89)	1.2 (5)
Study grant, % (*n*)	7.3 (54)	10.5 (64)	-	14.6 (59)
Parents, % (*n*)	8.1 (60)	7.4 (45)	0.2 (1)	10.4 (42)
Other, % (*n*)	13.7 (102)	4.0 (25)	9.5 (57)	3.5 (14)
Gross monthly income, *n*	677	567	559	391
No own income, % (*n*)	9.0 (61)	6.0 (34)	3.0 (17)	7.9 (31)
Minimal or below, % (*n*)	70.3 (476)	30.5 (173)	15.2 (85)	36.3 (142)
Above minimal, below modal, % (*n*)	16.2 (110)	35.1 (199)	34.5 (193)	28.1 (110)
Above modal, % (*n*)	4.4 (30)	28.4 (161)	47.2 (264)	27.6 (108)

Note. M, mean; SD, standard deviation; IQ, intelligence quotient, estimated based on the four WAIS subscales. ^1^ *n* is complete for this variable. ^2^ people with psychosis *n* = 920, siblings *n* = 870, parents *n* = 834, controls *n* = 492. ^a^ Living situation of parents was only assessed in Maastricht.

**Table 2:** Mixed-effects logistic regression on people with psychosis, siblings, and parents.

	Empty Model (*n* = 2703)		Multivariable Model (*n* = 1866)	
Predictors	OR [CI]	*p*	OR [CI]	*p*
Ethnicity: Caucasian	-	-	REF	REF
Non-Caucasian	-	-	1.65 [1.09, 2.50]	0.002
Education level: None	-	-	REF	REF
Practice-oriented	-	-	0.39 [0.06, 2.85]	0.19
Intermediate	-	-	0.19 [0.03, 1.38]	0.02
Theory-oriented	-	-	0.12 [0.02, 0.90]	0.004
Living situation: Single	-	-	REF	REF
Sheltered	-	-	0.93 [0.43, 1.90]	0.79
With parent(s)	-	-	0.63 [0.40, 0.97]	0.006
With partner/family	-	-	0.25 [0.14, 0.41]	<0.001
Other	-	-	1.03 [0.51, 2.00]	0.90
Familial random effect		<0.001		0.82
Variance	0.97		0.07	
SD	0.99		0.26	
ICC	0.23		0.02	

Note. OR, odds ratios; CI, 99% confidence interval; REF, reference category; SD, standard deviation; ICC, intraclass correlation coefficient.

**Table 3:** Regression analyses (step 1) and multiple regression analysis and bootstrap (step 2) for unmet financial needs in people with psychosis.

	Sample A (*n* = 478)				Sample B (*n* = 478)				Total Sample (*n* = 956)				
	Step 1: Simple Linear Regression								Step 2: Multiple Regression; Bootstrap				
										99% CI			
Predictors	β	SE	*p*	R^2^	β	SE	*p*	R^2^	β	Lower	Upper	SE	*p*
Demographic characteristics													
Age	0.02	0.01	NS	<0.01									
Gender	0.30	0.19	NS	0.01									
Region of assessment				0.03				0.01					
Utrecht	REF	REF	REF		REF	REF	REF						
Amsterdam	0.70	0.24	0.003 *		0.22	0.24	NS						
Groningen	0.80	0.24	<0.001 *		0.26	0.24	NS						
Maastricht	0.85	0.26	<0.001 *		0.41	0.24	NS						
Education level				0.06									
None	REF	REF	REF										
Practice-oriented	−0.93	0.82	NS										
Intermediate	−1.76	0.82	NS										
Theory-oriented	−1.84	0.84	NS										
Marital status	−0.80	0.29	0.006 *	0.02	0.62	0.28	NS	0.01					
Living situation				0.02									
Single	REF	REF	REF										
With parent(s)	−0.16	0.20	NS										
With partner/family	−0.33	0.30	NS										
Sheltered	0.25	0.32	NS										
Other	0.67	0.35	NS										
Economic characteristics													
Source of income				0.05				0.04					
Other	REF	REF	REF		REF	REF	REF						
Wages	−1.16	0.32	<0.001 *		−0.48	0.32	NS						
Benefits—pension	−1.56	0.63	NS										
Benefits—illness invalidity	−0.58	0.30	NS										
Benefits—unemployment	−0.47	0.38	NS										
Study grant	−0.97	0.42	NS										
Parents	−0.92	0.45	NS										
Gross monthly income				0.02									
No own income	REF	REF	REF										
Minimal or below	0.04	0.34	NS										
Above minimal, below modal	−0.58	0.40	NS										
Above modal	−0.42	0.58	NS										
Psychiatric characteristics													
Illness duration	0.00	0.02	NS	<0.001									
Number of psychotic episodes	0.16	0.07	NS	0.01									
PANSS positive symptoms	0.05	0.01	<0.001 *	0.03	0.05	0.01	<0.001 *	0.04	0.03	−0.004	0.06	0.01	NS
PANSS negative symptoms	0.01	0.01	NS	<0.01									
Being in remission	0.27	0.17	NS	0.01									
CAN proportion of unmet needs	0.57	0.29	NS	0.01									
Functional characteristics													
GAF symptoms	−0.02	0.01	<0.001 *	0.03	−0.02	0.01	0.001 *	0.03	−0.01	−0.02	0.02	0.01	NS
GAF disabilities	−0.02	0.01	<0.001 *	0.03	−0.02	0.01	<0.001 *	0.05	−0.01	−0.03	0.01	0.01	NS
Substance use													
Tobacco units per day	0.04	0.01	<0.001 *	0.05	0.04	0.01	<0.001 *	0.06	0.03	0.02	0.05	0.01	<0.001 *
Alcohol units per week	−0.00	0.01	NS	<0.001									
Cannabis use last 12 months				0.05				0.08					
None	REF	REF	REF		REF	REF	REF		REF	REF	REF	REF	REF
Less than weekly	0.83	0.29	0.004 *		0.89	0.30	0.003 *		0.57	−0.08	1.22	0.24	NS
Weekly	0.41	0.30	NS										
Daily	0.87	0.21	<0.001 *		1.16	0.24	<0.001 *		0.54	0.03	1.07	0.19	0.003 *
Lifetime other drugs use	−0.37	0.17	NS	0.01									
Cognitive functioning													
Composite score	0.13	0.06	NS	0.02									

Note. * Significant if *p* < 0.01, CI, confidence interval; SE, standard error; NS, non-significant; REF, reference category; PANSS, Positive And Negative Syndrome Scale; CAN, Camberwell Assessment of Need; GAF, Global Assessment of Functioning.

## Data Availability

Under the General Data Protection Regulation (GDPR), our data are considered pseudonymized rather than anonymized and are therefore still regarded as personal data. Given that participants have not given informed consent to have their personal data publicly shared, we are legally and ethically not allowed to publish our dataset. Data are therefore only available upon request via an application form to the GROUP committee via email.

## Supplementary Materials

The following supporting information can be downloaded at: https://www.mdpi.com/article/10.3390/jcm13195945/s1, Figure S1: STROBE flowchart: Strengthening the Reporting of Observational Studies in Epidemiology; Table S1: Characteristics of people with psychotic disorders (n = 956) and correlations with unmet financial needs; Table S2: Meeting financial needs of people with psychosis, siblings, parents, and controls per assessment.
