# Supplementary material for: Unmet Financial Needs of People with Psychotic Disorders—A Cross-Sectional Study in People with Psychotic Disorders, Parents, Siblings, and Controls
Source: J Clin Med. 2024 Oct 6;13(19):5945. doi: 10.3390/jcm13195945 (PMC11477704; doi:10.3390/jcm13195945)
Supplement: Supplementary file 1 [file jcm-13-05945-s001.zip › jcm-3207465-supplementary.pdf]

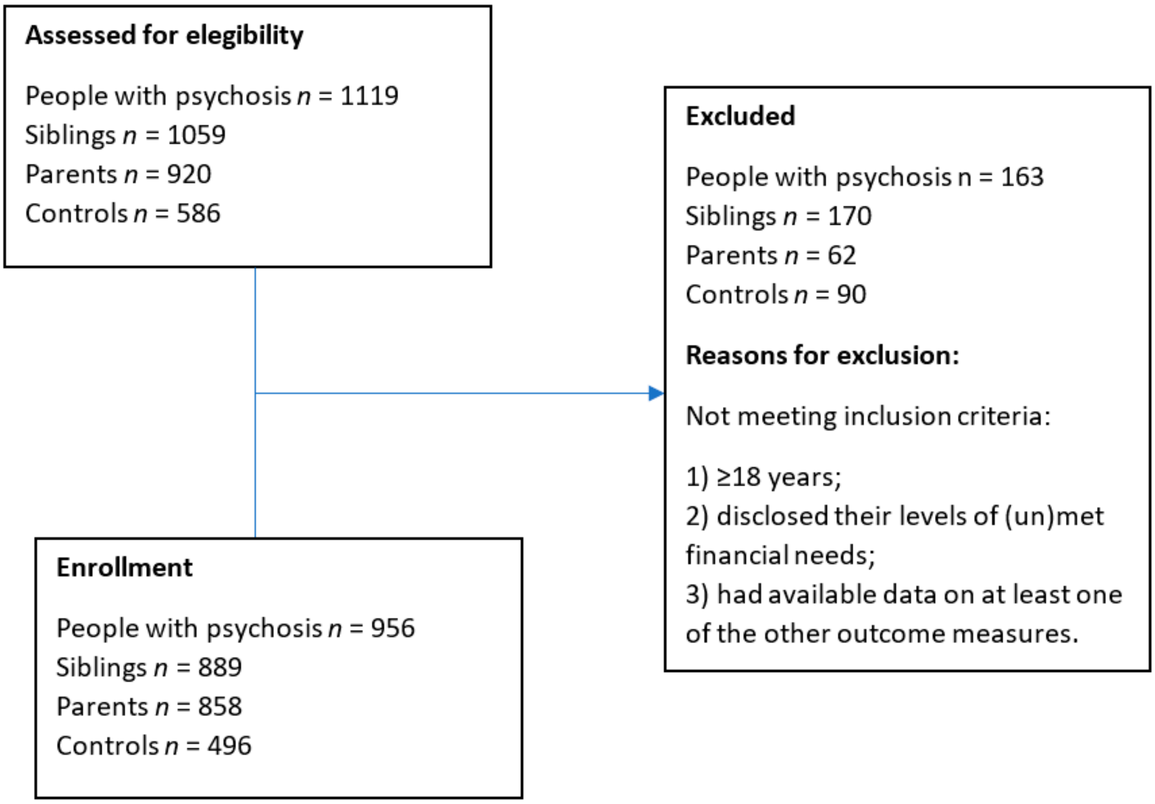

Figure S1. STROBE flowchart: Strengthening the Reporting of Observational Studies in Epidemiology.

Table S1. Characteristics of people with psychotic disorders (n = 956) and correlations with unmet financial needs.

	n	M (SD)	% (n)	ρ [99% CI]
Age	956	28.2 (7.9)		-0.02 [-0.11 -0 .06]
Psychiatric characteristics				
Diagnosis				
Schizophrenia			66.5 (628)	
Schizo-affective disorder			11.2 (106)	
Schizophreniform disorder			5.5 (52)	
Delusional disorder			1.7 (16)	
Substance induced psychosis			0.4 (4)	
Bipolar disorder with psychotic features			0.7 (7)	
Brief psychotic disorder			3.0 (28)	
Psychosis NOS			10.6 (100)	
Other			0.4 (4)	
Illness duration (years)	921	4.8 (4.6)		0.005 [-0.08 - 0.09]
Number of psychotic episodes	923	1.7 (1.1)		-0.002 [-0.09 - 0.09]
PANSS positive symptoms	894	13.8 (6.6)		-0.19* [-0.27 - -0.10]
PANSS negative symptoms	888	15.0 (6.6)		-0.08 [-0.17 - 0.01]
Being in remission (yes)	907		46.2 (419)	
CAN proportion of unmet needs	866	0.4 (0.3)		-0.07 [-0.16 – 0.02]
Functional characteristics				
GAF symptoms	851	56.1 (15.9)		0.16* [0.07 - 0.25]
GAF disabilities	851	54.3 (16.3)		0.20* [0.11 - 0.28]
Substance use				
Tobacco units per day	951	11.7 (10.9)		-0.24* [-0.32 - -0.16]

Alcohol units per week	960	6.4 (10.5)		-0.02 [-0.11 - 0.07]
Cannabis use last 12 months	951			
None			62.9 (598)	
Less than weekly			9.3 (88)	
Weekly			9.4 (89)	
Daily			18.5 (176)	
Lifetime other drugs use (yes)	956		39.7 (380)	
Cognitive characteristics				
Composite score	757	0.0 (1.7)		0.14* [0.04 - 0.23]

Note. M, mean; SD, standard deviation; ρ, Spearman Rho correlation; CI, confidence interval; NOS, not otherwise specified; PANSS, Positive And Negative Syndrome Scale; CAN, Camberwell Assessment of Need, GAF, Global Assessment of Functioning. *Significant at p <0.01.

Table S2. Meeting financial needs of people with psychosis, siblings, parents, and controls per assessment.

		**People with psychosis**	**Siblings**	**Parents**	**Controls**
	n	956	889	858	496
1st assessment	Mean (SD)	3.2 (1.2)	3.8 (1.0)	4.0 (0.9)	3.9 (0.9)
	Unmet financial needs, total, % (n)	24.0 (230)	8.4 (74)	4.1 (35)	7.9 (39)
	n	741	777	-	446
2nd assessment[1]	Mean (SD)	3.5 (1.1)	4.0 (0.9)	-	4.1 (0.8)
	Unmet financial needs, total, % (n)	16.7 (124)	4.5 (35)	-	3.6 (16)
	n	593	669	-	374
3rd assessment[2]	Mean (SD)	3.5 (1.1)	3.9 (0.9)	-	4.1 (0.8)
	Unmet financial needs, total, % (n)	16.7 (99)	5.7 (38)	-	2.2 (8)

Note. M, mean; SD, standard deviation. 1 3-year follow-up. 2 6-year follow-up.
